# In Vitro Assessment of Artificial Aging on the Antifungal Activity of PMMA Denture Base Material Modified with ZrO_2_ Nanoparticles

**DOI:** 10.1155/2021/5560443

**Published:** 2021-05-13

**Authors:** Shorouq Khalid Hamid, Lujain Ali Alghamdi, Faris A. Alshahrani, Soban Q. Khan, Asif Matin, Mohammed M. Gad

**Affiliations:** ^1^College of Dentistry, Imam Abdulrahman Bin Faisal University, P.O. Box 1982, Dammam 41441, Saudi Arabia; ^2^Department of Substitutive Dental Sciences, College of Dentistry, Imam Abdulrahman Bin Faisal University, P.O. Box 1982, Dammam 41441, Saudi Arabia; ^3^Department of Dental Education, College of Dentistry, Imam Abdulrahman Bin Faisal University, P.O. Box 1982, Dammam 41441, Saudi Arabia; ^4^IRC-Membranes & Water Security, King Fahd University of Petroleum & Minerals, Dhahran 31261, Saudi Arabia

## Abstract

The antifungal effect of zirconium dioxide nanoparticles (ZrO_2_NPs) incorporated into denture base material has been inadequately investigated; additionally, to the authors' knowledge, no studies have assessed the influence of artificial aging on the antifungal activity of these particles. *Methodology*. Heat-polymerized acrylic resin disks were fabricated and divided into four groups (0%, 1%, 2.5%, and 5% ZrO_2_NPs by weight). Antifungal activity was assessed using the direct culture and disk diffusion methods. Surface roughness and contact angles were measured using a profilometer and a goniometer, respectively. The artificial aging procedure was performed by repeating all tests at 7, 14, and 30 days following 2 rounds of thermocycling. Data were analyzed using ANOVA and Tukey's post-hoc test (*p* < 0.05). *Results*. The addition of ZrO_2_NPs significantly decreased the adhesion of *Candida albicans* with and without artificial aging procedures (*p* < 0.001), while the disk diffusion methods did not reveal inhibition zones. ZrO_2_NP-modified specimens displayed significantly higher surface roughness compared to specimens in the control group (*p* < 0.05) and showed the same behaviors with artificial aging procedures. The contact angle was significantly decreased in all modified groups in comparison to the control group (*p* < 0.05). *Conclusion*. The addition of ZrO_2_NPs to polymethylmethacrylate denture base material reduced the adhesion of *Candida albicans* with a long-term antifungal effect. With the addition of ZrO_2_NPs, contact angles were decreased and surface roughness was increased; 1% was the most appropriate concentration. *Clinical significance.* The addition of ZrO_2_NPs to denture base material confers a long-term antifungal effect and could be used as a possible method for preventing and treating denture stomatitis.

## 1. Introduction

Denture-induced stomatitis (DIS) is a widespread disease affecting 65% of denture wearers, with the most affected site being the palatal mucosa [1]. Several factors have been implicated in the development of DIS, including decreased salivary flow, ill-fitting dentures, poor oral hygiene, trauma to the oral mucosa, and microbial infections, primarily fungal infections with *Candida albicans* (*C. albicans*) [2]. Many studies have investigated the role of *C. albicans*, yielding strong evidence that it is the primary fungal source [3, 4]. Additionally, denture base material is naturally porous, rendering its surface a favorable medium for the adherence and colonization of *C. albicans* [5]. Subsequently, biofilm formation occurs, which decreases cleansing efficacy and increases resistance to antifungal treatment [6].

Several treatment modalities have been fabricated to prevent fungal growth on denture bases, including the use of different denture cleansing agents [7] and antifungal medications [8] as well as the incorporation of antifungal agents into denture base materials [9]. However, using denture cleansing agents could cause deterioration of the denture base and lead to a rougher surface, consequently increasing susceptibility to *Candida* adhesion [10]. Moreover, the therapeutic failure of antifungal medications is common due to the diluent effect of saliva, the cleaning action of oral muscles, and the dependence on patient compliance [8].

Various studies have shown tremendous advances in nanomaterials. These materials have demonstrated numerous promising applications in the fields of biomedical science and nanomedicine [11] due to their excellent chemical, physical, and antifungal properties [12]. In particular, zirconium dioxide nanoparticles (ZrO_2_NPs) have received increasing attention for their technological and scientific features [13]. A 2018 study evaluated the effect of the incorporation of ZrO_2_NPs into heat-polymerized polymethylmethacrylate (PMMA) denture base material in different concentrations (2.5%, 5%, and 7.5% ZrO_2_NPs by weight). It was concluded that the addition of ZrO_2_NPs to the denture base resin material increased the tensile strength of these resins [14]. Additionally, Ahmed et al. [15] found that the incorporation of ZrO_2_NPs into heat-polymerized denture base material in concentrations of 1.5%, 3%, 5%, and 7% by weight increased the material's flexural strength, hardness, and fracture toughness, especially at the highest concentration.

It is essential to anticipate the natural effects of aging on material properties. The wet environment and temperature fluctuation of the oral cavity further affect denture base properties [16]. Therefore, an artificial aging procedure is used to mimic oral cavity conditions by way of thermocycling; tested material is subjected to alternating extreme temperatures [17]. A total of approximately 10,000 cycles in a thermocycling machine are considered equal to 1 year of clinical use [16, 18].

Gowri et al. [13] assessed the antifungal activity of ZrO_2_NPs and found that they significantly inhibited the growth of *Aspergillus niger* and *C. albicans* strains. A 2017 study took a new approach to preventing DIS by evaluating the inhibitory effect of ZrO_2_NPs as an antifungal agent against *C. albicans* adhesion in heat-polymerized PMMA denture base material repaired with autopolymerized acrylic resin. That study demonstrated that ZrO_2_NPs had a significant inhibitory effect against *C. albicans* adhesion and that a denture base modified with ZrO_2_NPs could be used as a method for DIS prevention [19].

Previous studies have examined the antifungal activity of ZrO_2_NPs, but their assessments were limited due to the absence of artificial aging procedures and the lack of surface property evaluations [13, 19]. Therefore, the antifungal effect of ZrO_2_NPs incorporated into denture base materials has been inadequately investigated. Additionally, to the authors' knowledge, no studies have yet evaluated the influence of artificial aging on the antifungal effects of ZrO_2_NPs. Hence, the current study was the first to assess such effects in correlation with the surface roughness and contact angle of ZrO_2_NP-modified PMMA denture base material.

The present study had two null hypotheses. The first was that the addition of ZrO_2_NPs to PMMA denture base material would have no long-term antifungal effects. The second was that the addition of ZrO_2_NPs to PMMA denture base material would have no effect on either surface roughness or contact angle.

## 2. Materials and Methods

### 2.1. Specimen Preparation

Sample size calculation showed that the necessary sample size at 80% power with a 5% level of significance and a 95% confidence interval was 160 specimens (10 per test). The specimens were distributed into 4 groups based on the concentration of ZrO_2_NPs (*n* = 40 for each): a control group with no additives and 3 modified groups with 1%, 2.5%, and 5% ZrO_2_NPs, respectively, by weight. ZrO_2_NPs (99.9% < 100 nm, 1314-23-4; Shanghai Richem International Co., Ltd., Shanghai, China) were added in concentrations of 1%, 2.5%, and 5% by weight to heat-polymerized acrylic resin powder (BMS 014 powder; BMS Dental, Capannoli, PI, Italy). In order to improve adhesion between ZrO_2_NPs and the acrylic resin matrix, a silane coupling agent (3-(trimethoxysilyl) propyl methacrylate) (TMSPM) (Shanghai Richem International Co., Ltd., Shanghai, China) was utilized to treat the ZrO_2_NPs. Additionally, 0.3 g of TMSPM was dissolved in 100 mL of acetone in order to confirm that the ZrO_2_NPs were uniformly coated. Using a magnetic stirrer (Cimarec Digital Stirring Hotplates, SP131320-33Q; Thermo Fischer Scientific, Waltham, MA, USA), 30 g of ZrO_2_NPs was added to the TMSPM/acetone solution and stirred for 1 hour. Next, to eliminate the solvent, a rotary evaporator under a vacuum was utilized for 30 minutes at 60°C and 150 rpm. In order to obtain the treated ZrO_2_NPs, samples were dried, subjected to 120°C for 2 hours, and then bench-cooled [19–21]. The treated ZrO_2_NPs were weighed using an electronic balance (S-234; Denver Instrument, Gottingen, Germany) and incorporated into a heat-polymerized acrylic resin powder and then mixed using a mortar and pestle. Subsequently, these samples were stirred for 30 minutes to ensure an even particle distribution.

Disk-shaped, heat-polymerized acrylic resin specimens with dimensions of 10 × 2 ± 0.1 mm were fabricated following the conventional method and steps for denture base fabrication in accordance with the manufacturer's instructions [16, 20, 21]. Wax specimens (Vertex Dental B. V., Soesterberg, Netherlands) were obtained using negative molds and then invested in metal flasks (61B Two Flask Compress; Handler Manufacturing) and type III dental stones (Fujirock EP; GC Corporation, Tokyo, Japan). Next, the wax was eliminated with the aid of a wax elimination machine to obtain the negative mold spaces. While the stones were still warm, the separating medium (162-800-00; Vandex Isoliermittel GmbH, Hamburg, Germany) was spread over the stones' surfaces. A water/powder ratio was measured and then mixed in accordance with the manufacturer's instructions. The mixture was packed at the dough stage into the previously created molds under pressure. Next, the flasks were kept in flask clamps for 1 hour and then processed using a conventional heat curing unit (KaVo Elektrotechnisches Werk GmbH, Biberach, Germany) at 74°C for 8 hours, then at 100°C for 1 hour. Following polymerization, the flasks were cooled to room temperature and then deflasked.

After deflasking, the excess resins from the deflasked specimens were removed and finished with a tungsten carbide bur (HM 79GX-040 HP; Meisinger USA, Centennial, CO, USA) with a thin cross-cut at 18,000 rpm. Next, the specimens were polished through a coarse-grain and then a fine-grain cylindrical rubber top bur (Super Acrylic Polisher; Long Dental). Polishing occurred only on the specimens' cameo surfaces, with the intaglio surfaces remaining unpolished. The specimens' dimensions were verified using a digital caliper with an accuracy of 0.01 mm (Neiko 01407A Electronic Digital Caliper). Specimens were kept at 37°C in distilled water for 1 week. To minimize the accumulation of residual monomers, the distilled water was changed daily.

### 2.2. Aging Procedures

**Figure 1:**
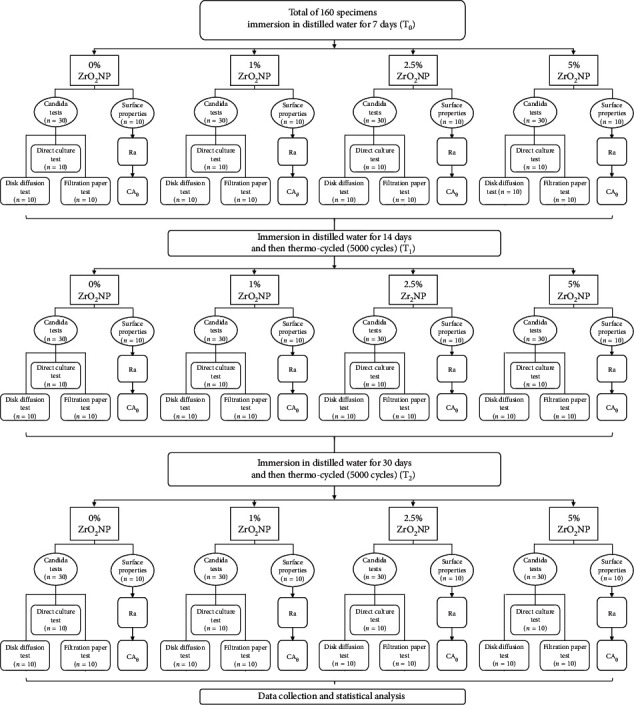
Flow diagram to illustrate study design. (CA) *Candida* adhesion; (Ra) surface roughness; (CA_*θ*_) contact angle; (T_0_, T_1_, T_2_) time intervals of 7, 14, and 30 days, respectively.

All specimens were marked numerically for standardized testing before, during, and after aging procedures. Next, baseline readings were taken for antifungal activity, surface roughness, and contact angle tests (T_0_). All specimens were subsequently water-immersed for 2 weeks, after which they were subjected to 5,000 cycles in a thermocycling machine (Thermocycler THE-1100; SD Mechatronik GmbH, Feldkirchen-Westerham, Germany) between temperatures of 5°C and 55°C, with a dwell time of 30 seconds for each specimen [17]. Antifungal activity, surface roughness, and contact angle tests were then repeated (T_1_). All specimens were then immersed in water for 30 days, after which they were subjected to an additional 5,000 cycles to ultimately simulate 1 year of clinical use [16]. Lastly, all tests were repeated (T_2_) to assess the long-term antifungal efficacy of the treatment (Figure 1).

### 2.3. Candida Adherence Assay

Prior to the assessment of antifungal properties, the specimens were submerged in 70% alcohol for 20 minutes and then ultrasonically cleaned using sterilized distilled water [22]. The specimens were then immersed in artificial saliva, composed of mucin, methyl-4-hydroxybenzoate, benzalkonium chloride, ethylenediaminetetraacetic acid (EDTA), H_2_O_2_, xylitol, peppermint oil, spearmint oil, and mineral salts (A.S. Orthana, Biofac A/S, Kastrup, Denmark), with 2,000,000 *C. albicans* cells (ATCC 10231) for 2 days at 37°C. The specimens were then rinsed 3 times using phosphate-buffered saline (PBS, pH 7.2) to remove nonadherent cells. Subsequently, the specimens were inserted into sterile tubes that each contained 1 mL of Sabouraud dextrose broth (Acu Medica Lab Systems Ltd., Mumbai, Maharashtra, India) for 1 day. The specimens were then vortexed for 10 minutes, after which they were centrifuged for 5 minutes at 4,500 rpm. Then, the specimens were removed from the tubes, leaving the clustered pellets behind in the tubes. Lastly, the *C. albicans* colonies that adhered to each specimen were calculated using the direct culture test [19, 20].

### 2.4. Direct Culture Method (Colony-Forming Units, CFU)

A 10 *μ*L sample was taken from each centrifuge tube. Then, the samples were serially diluted and spread onto petri dishes with Sabouraud dextrose agar (LabM, Lancashire, England), after which they were incubated for 1 day at 37°C. The number of *C. albicans* colonies in every quadrant was next measured with the aid of a colony counter pen (SP Scienceware; Bel-Art Products Inc., Pequannock, NJ, USA) where noticeable growth was observed, and the final numbers of colonies were corrected for the dilution factor [19, 20].

### 2.5. Agar Disk Diffusion and Filtration Paper Methods

Specimens were placed on blood agar plates and incubated at room temperature for 120 minutes for the diffusion of the antifungal agents [23]. Additionally, the filtration paper disk diffusion method was performed, and the acrylic disks were immersed in distilled water for 48 hours. Drops of the emerging solution were added to the sterile filter paper disks (10 mm in diameter) and then placed on the surfaces of blood agar plates [24]. Then, the disk diffusion and filtration paper plates were anaerobically incubated for 1 day at 37°C. The presence of fungal growth inhibition zones around the disks and filtration papers was visually inspected.

### 2.6. Surface Roughness (Ra)

To determine the surface roughness (Ra) value, a noncontact optical interferometric profilometer (Contour GT; Bruker Nano GmbH, Berlin, Germany) was employed at a 0.01 mm resolution. Specimens were scanned for an approximate area of 0.43 × 0.58 mm using a standard camera at 20× magnification at 5 sites of each specimen. Then, the average calculation was performed. Analysis of the obtained images was conducted by a software package (Vision64, Bruker Nano) to assess pit features and to calculate the Ra values of all specimens [20].

### 2.7. Contact Angle Measurement

The contact angle measurement was conducted using the sessile drop test. First, the specimens' surfaces were gently air-dried. Then, distilled water droplets were placed on the specimens' surfaces using an autopipette and a goniometer in order to standardize the volume of the droplets (2 *μ*L each). The contact angles were measured using an automated contact angle goniometer (DM-501; Kyowa Interface Science Co, Japan). The tangent angle to the surface of the water droplet was measured and then remeasured 4 times on various sites of each specimen. Then, the average angles were calculated, and the images were analyzed using FAMAS software (Kyowa Interface Science Co., Japan) [20].

### 2.8. Statistical Analysis

Data analysis was performed using IBM SPSS Statistics for Windows version 19.0 (IBM Corp., Armonk, NY, USA). A Shapiro-Wilk test was performed to evaluate the normality of the data, after which parametric tests were conducted, generating significant results for the normality test. For inferential statistics, ANOVA was utilized to determine total significance in the tested variables (*Candida* count, Ra, and contact angle) in comparison with time and concentration. Furthermore, Tukey's post-hoc was used for pairwise comparison. For the combined effect of time and concentration, a 2-way ANOVA was performed. A *p* value of ≤0.05 was considered statistically significant.

## 3. Results

### 3.1. Antifungal Activity Assessment

**Figure 2:**
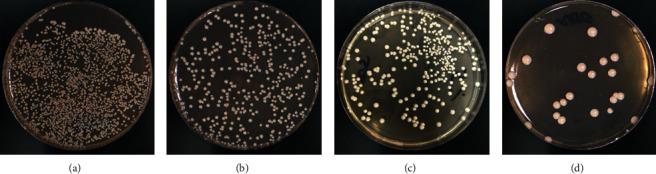
Direct culture method based on different ZrO_2_NP concentrations of heat-polymerized acrylic resin. (a) Control; (b) 1% ZrO_2_NPs; (c) 2.5% ZrO_2_NPs; (d) 5% ZrO_2_NPs.

Results of the direct culture method showed that the ZrO_2_NP-modified groups displayed significant antifungal effects for all tested time intervals compared to the control group (*p* < 0.001). Furthermore, the number of *C. albicans* colonies significantly decreased as the ZrO_2_NP concentration increased (*p* < 0.05). All of the pairwise comparisons exhibited significant differences between the means, except between the 2.5% and 5% groups in T1 (Table 1) (Figure 2). Variation across time intervals was examined for all concentrations. For the ZrO_2_NP-modified groups, there were significant antifungal effects (*p* < 0.001) between all the time intervals except between T0 and T2 in the 1% group and between T0 and T1 in the 5% group (Table 2). However, the disk diffusion and filtration paper methods showed negative results (absence of inhibition zones) for ZrO_2_NP-modified groups in all the tested time intervals.

### 3.2. Surface Roughness (Ra)

**Figure 3:**
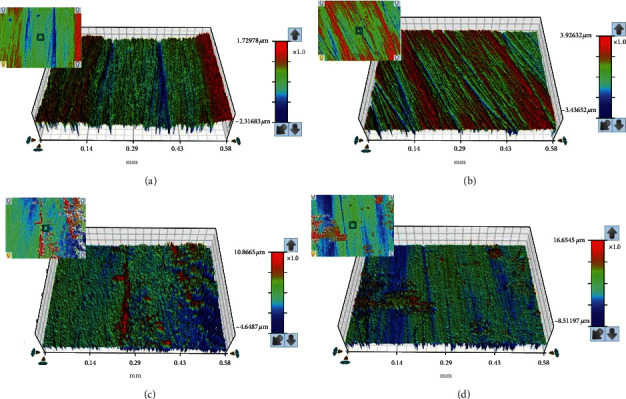
Representative surface roughness images of tested specimens based on different ZrO_2_NP concentrations of heat-polymerized acrylic resin. (a) Control; (b) 1% ZrO_2_NPs; (c) 2.5% ZrO_2_NPs; (d) 5% ZrO_2_NPs.

A statistically significant increase in Ra was found in relation to higher concentrations of ZrO_2_NPs added to the heat-polymerized acrylic resin compared to the control group at all time intervals (*p* < 0.001) (Table 1). The mean Ra values measured between time intervals at a given concentration were found to be statistically insignificant for the 1% and 2.5% groups (*p* < 0.05). Meanwhile, the 0% and 5% groups exhibited a significant difference (*p* < 0.05), and the pairwise comparison showed an insignificant difference only of T1 when compared to T0 and of T2 when compared to T1 in both the 0% and 5% ZrO_2_NP-modified groups (Table 2) (Figure 3).

### 3.3. Contact Angle

**Figure 4:**
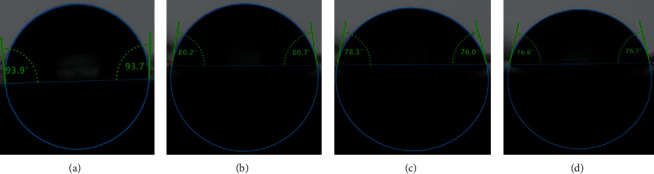
Contact angle measurements of tested specimen surfaces based on different ZrO_2_NP concentrations of heat-polymerized acrylic resin. (a) Control; (b) 1% ZrO_2_NPs; (c) 2.5% ZrO_2_NPs; (d) 5% ZrO_2_NPs.

A significant reduction (*p* < 0.05) was found in contact angle measurement in all of the ZrO_2_NP-modified groups in comparison to the control group at all time intervals, as shown in Table 1 and Figure 4. In comparison to the time interval at a given concentration, all groups displayed significant differences (*p* < 0.05) except the 1% and 5% test groups (Table 2).

## 4. Discussion

A denture base with antifungal activity might be used as a method to prevent DIS and could help denture users who have disabilities or difficulties with appropriate denture cleaning [20]. An ideal criterion for any antimicrobial agent is that it should have long-term antimicrobial activity [25]. However, no previous studies have investigated the influence of artificial aging on the antifungal activity of heat-polymerized acrylic resin denture bases modified with ZrO_2_NPs. Therefore, the current study aimed to investigate the influence of artificial aging on the antifungal activity of ZrO_2_NPs in correlation with the Ra and contact angle of heat-polymerized acrylic resin. The results demonstrated a significant long-term reduction in the adhesion of *C. albicans* in denture bases modified with ZrO_2_NPs. Furthermore, the addition of ZrO_2_NPs decreased the contact angle while increasing Ra. Therefore, the two null hypothesis of the present study (that the heat-polymerized acrylic resin modified with ZrO_2_NPs would have no long-term antifungal activity and no effect on Ra or contact angle) were rejected.

The associations between ZrO_2_NP concentrations and the numbers of *C. albicans* colonies demonstrated the antifungal activity of ZrO_2_NPs, a finding consistent with those of previous studies [13,19]. In the present study, long-term antifungal activity was investigated by repeating all the tests at different time intervals. The results showed that a heat-polymerized acrylic resin denture base material modified with ZrO_2_NPs demonstrated durable antifungal activity after undergoing an artificial aging procedure that simulated 1 year of clinical use.

In the present study, the results of the direct culture method indicated a significant reduction in the number of *C. albicans* colonies in all ZrO_2_NP-modified groups. The lowest number of *C. albicans* colonies was observed in the 5% ZrO_2_NP group. This reduction in *C. albicans* colonies could be the result of the antimicrobial activity of ZrO_2_NPs or the enhancement of denture base surface properties [19]. Another study suggested that the antimicrobial effect of nanoparticles is due to the chemical reactivity of nanoparticle crystals that might significantly be influenced by their shape, which is affected by the arrangement of surface atoms, surface energy, and bonding [26]. Various atomic planes exhibit different surface energies, and the surface energy of nanostructures is primarily affected by the density of the dangling bonds present on their surfaces. The difference in surface energy is crucial in determining the antifungal activity of nanoparticles [27]. Furthermore, it might be presumed that ZrO_2_NPs have identical surface geometries, but with various shapes, several active planes could exhibit different antimicrobial effects [11]. Another explanation for the antifungal activity of ZrO_2_NPs is the presence of some nanoparticles on the specimens' surfaces, which might lead to direct contact between the nanoparticles and *C*. *albicans.* This could, in turn, actively prevent the growth of fungal strains by interrupting cell function, thereby deforming the fungal hyphae [13].

The results of both the conventional and filtration paper disk diffusion techniques revealed the absence of inhibition zones for all the tested concentrations of ZrO_2_NPs. This negates the theory that the mechanism of the antifungal activity of ZrO_2_NPs is the leaching of nanoparticle ions. The antifungal activity of ZrO_2_NPs could be the result of their effect on PMMA denture base surface properties, and as such, further investigations should be conducted to confirm the antifungal mechanism of ZrO_2_NPs.

Adhesion of *C. albicans* has been shown to directly correlate with Ra, owing to the ability of *C. albicans*, in its mycelial form, to infiltrate the minuscule protrusions on the rough surface of the denture base [28]. The results of the present study revealed significant increases in Ra with higher concentrations of ZrO_2_NPs and exhibited the same behaviors with respect to aging procedures. However, a more than 1% addition of ZrO_2_NPs increased Ra beyond the acceptable clinical value (0.2 *μ*m) [5]. This increase might be attributable to the agglomeration and cluster formation of nanoparticles on the specimens' surfaces [29]. Also, previous studies have reported that Ra increases with higher concentrations of nanoparticles [29, 30].

Similarly to the current study, Gad et al. [31] assessed the Ra of a heat-polymerized acrylic resin denture base material modified with ZrO_2_NPs at concentrations of 0.5%, 1%, 2.5%, and 5%. Their results demonstrated a significant increase in Ra beyond the acceptable clinical value (0.33–0.49 *μ*m) in modified groups except for the 0.5% group (0.15 *μ*m). The authors related this increase in Ra for higher ZrO_2_NP concentrations to the clustering liability of nanoparticles as well as to the reduced homogeneity of the resin matrix, which was observed to be concentration-dependent. Furthermore, Lee et al. [32] found a significant increase in Ra with the incorporation of nanographene oxide into PMMA resin, which was mostly the result of exposed nanographene oxide on the outer surface after polishing. However, according to Fouda et al. [20], the addition of nanodiamonds in concentrations of 0.5%, 1%, and 1.5% to a heat-polymerized acrylic resin denture base material decreased Ra. This might be attributable to the small size of the particles, which decreases the interparticle distance, leading to proximate contact between the nanoparticles at lower concentrations.

One of the major factors affecting the adhesion of *C. albicans* is surface hydrophobicity. The cell surface of *C. albicans* is hydrophobic in nature, especially in its hyphal form, which improves its adherence to hydrophobic acrylic surfaces through the hydrophobic interaction [33]. Hydrophobic interactions occur between the cell surface and the substratum, which allows the cell to overcome the repulsive forces active within a specific distance from the substratum surface, although this depends on the surface hydrophobicity of the microbes [34]. Therefore, increasing surface hydrophilicity would represent an effective method for decreasing *C. albicans* adhesion [35].

According to the results of the present study, ZrO_2_NP modification yielded a significant decrease in contact angle. This reduction, in turn, indicates an increase in surface hydrophilicity. This finding might be explained by the interaction between H_2_O and ZrO_2_NPs, which culminates in both molecular and dissociative adsorption of water [35], forming several polar interactions and, consequently, hydrophilic hydroxyl (−OH) species on the surfaces of the particles [36]. According to Lee et al. [32], the addition of nanographene oxide to PMMA increases surface hydrophilicity due to its ability to create a hydration layer on the surface and produce a tightly bound water layer that represents a physical barrier to prevent adhesion of *C. albicans*.

Although Ra increased with ZrO_2_NP addition in the present study, numbers of *C. albicans* significantly decreased, confirming the strong antifungal effect of ZrO_2_NPs. On the other hand, this effect could be the result of surface changes from decreasing the contact angle, thus increasing surface hydrophilicity and hence reducing *C. albicans* adhesion. Additionally, ZrO_2_NPs enhance the mechanical performance of PMMA denture base materials [14,15]. Clinically, ZrO_2_NPs can be incorporated into PMMA due to its durability with respect to decreasing the adhesion of *C. albicans*. The durability of the antifungal activity of the heat-polymerized acrylic resin denture base material modified with ZrO_2_NPs would promote denture hygiene, particularly among patients with physical disabilities. Additional studies should be conducted to evaluate the mechanism of the antifungal activity of ZrO_2_NPs.

One strength of the present study was that it applied 3 techniques for assessing the long-term antifungal activity of heat-polymerized acrylic resin modified with ZrO_2_NPs. Additionally, an artificial aging procedure (thermocycling) was conducted to simulate 1 year of clinical use. However, the analysis was limited in that it evaluated the specimens in settings that differ from the oral cavity environment with regard to alterations in temperature and pH and the absence of natural saliva, which contains a variety of species of microorganisms. Nanoparticle-modified denture base material displayed improved physical and mechanical properties in the present study, but further research investigating the addition of nanoparticles to computer-aided design and computer-aided manufacturing (CAD/CAM) prepolymerized disks should be performed in order to solidify this method as a promising technique [37].

## 5. Conclusions

Despite the limitations of the present analysis, we reached the following conclusions:

1. The incorporation of ZrO_2_NPs into heat-polymerized acrylic resin decreased *C. albicans* adhesion and conferred a long-term antifungal effect.
2. Adding ZrO_2_NPs to heat-polymerized acrylic resin decreased the contact angle and increased the surface roughness; 1% ZrO_2_NPs by weight were considered the most appropriate concentration.

## Figures and Tables

**Table 1:** Means, standard deviations, and *p*-values for heat-polymerized specimens modified with different concentrations of ZrO_2_NPs according to time.

Time	Concentration of ZrO_2_NPs (%)	Candida count (CFU)		Surface roughness (*μ*m)		Contact angle (°)	
		Mean (SD)	*p*-value	Mean (SD)	*p*-value	Mean (SD)	*p*-value
*T*_0_	0	2069.4 (60.9)	0.000^*∗*^	0.15 (0.01)	0.000^*∗*^	92.98 (1.1)	0.000^*∗*^
	1	1085.6 (42.6)		0.19 (0.01)		80.74 (1.3)^a^	
	2.5	781.8 (13.6)		0.23 (0.01)		79.39 (1.2)^a^	
	5	384.1 (13.0)		0.25 (0.01)		77.39 (1.4)	

*T*_1_	0	1971.5 (17.6)	0.000^*∗*^	0.16 (0.01)	0.000^*∗*^	91.27 (1.2)	0.000^*∗*^
	1	1429.1 (36.9)		0.19 (0.01)		80.15 (1.5)	
	2.5	655.2 (31.5)^a^		0.23 (0.01)		78.41 (1.4)^a^	
	5	379.3 (20.0)^a^		0.26 (0.01)		78.47 (1.4)^a^	

*T*_2_	0	1953.9 (30.2)	0.000^*∗*^	0.17 (0.01)	0.000^*∗*^	91.29 (1.1)	0.000^*∗*^
	1	1051.7 (28.8)		0.19 (0.01)		79.43 (1.2)^a^	
	2.5	584.1 (18.4)		0.23 (0.01)		77.62 (1.7)	
	5	317.2 (10.5)		0.27 (0.01)		77.87 (1.5)^a^	

Variation in concentration was studied at different time intervals vertically: (T_0_, T_1_, T_2_) of 7, 14, and 30 days, respectively. Same alphabets showed insignificant results in pairwise comparison. ^*∗*^(*p* > 0.05) was considered statistically insignificant.

**Table 2:** Means, standard deviations, and *p*-values for heat-polymerized specimens modified with different concentrations of ZrO_2_NPs according to concentration.

Concentration of ZrO_2_NPs (%)	Time	Candida count (CFU)		Surface roughness (*μ*m)		Contact angle (°)	
		Mean (SD)	*p*-value	Mean (SD)	*p*-value	Mean (SD)	*p*-value
0	T_0_	2069.4 (60.9)	0.000^*∗*^	0.15 (0.01)^a^	0.031^*∗*^	92.98 (1.1)	0.003^*∗*^
	T_1_	1971.5 (17.6)^a^		0.16 (0.01)^a,b^		91.27 (1.2)^a^	
	T_2_	1953.9 (30.2)^a^		0.17 (0.01)^b^		91.29 (1.1)^a^	

1	T_0_	1085.6 (42.6)^a^	0.002^*∗*^	0.19 (0.01)	0.648	80.74 (1.3)	0.103
	T_1_	1429.1 (36.9)		0.19 (0.01)		80.15 (1.5)	
	T_2_	1051.7 (28.8)^a^		0.19 (0.01)		79.43 (1.2)	

2.5	T_0_	781.8 (13.6)	0.000^*∗*^	0.23 (0.01)	0.258	79.39 (1.2)^a^	0.042^*∗*^
	T_1_	655.2 (31.5)		0.23 (0.01)		78.41 (1.4)^a,b^	
	T_2_	584.1 (18.4)		0.23 (0.01)		77.62 (1.7)^b^	

5	T_0_	384.1 (13.0)^a^	0.000^*∗*^	0.25 (0.01)^a^	0.004^*∗*^	77.39 (1.4)	0.255
	T_1_	379.3 (20.0)^a^		0.26 (0.01)^a,b^		78.47 (1.4)	
	T_2_	317.2 (10.5)		0.27 (0.01)^b^		77.87 (1.5)	

Variation in time was studied at different concentrations intervals vertically: (T_0_, T_1_, T_2_) of 7, 14, and 30 days, respectively. Same alphabets showed insignificant results in pairwise comparison. ^*∗*^(*p* > 0.05) was considered statistically insignificant.

## Data Availability

The data used to support this study are available from the corresponding author upon request.
